# Modeling the Cerebellar Microcircuit: New Strategies for a Long-Standing Issue

**DOI:** 10.3389/fncel.2016.00176

**Published:** 2016-07-08

**Authors:** Egidio D’Angelo, Alberto Antonietti, Stefano Casali, Claudia Casellato, Jesus A. Garrido, Niceto Rafael Luque, Lisa Mapelli, Stefano Masoli, Alessandra Pedrocchi, Francesca Prestori, Martina Francesca Rizza, Eduardo Ros

**Affiliations:** ^1^Department of Brain and Behavioral Sciences, University of PaviaPavia, Italy; ^2^Brain Connectivity Center, C. Mondino National Neurological InstitutePavia, Italy; ^3^NearLab - NeuroEngineering and Medical Robotics Laboratory, Department of Electronics, Information and Bioengineering, Politecnico di MilanoMilano, Italy; ^4^Department of Computer Architecture and Technology, University of GranadaGranada, Spain; ^5^Dipartimento di Informatica, Sistemistica e Comunicazione, Università degli Studi di Milano-BicoccaMilan, Italy

**Keywords:** cerebellum, cellular neurophysiology, microcircuit, computational modeling, motor learning, neural plasticity, spiking neural network, neurorobotics

## Abstract

The cerebellar microcircuit has been the work bench for theoretical and computational modeling since the beginning of neuroscientific research. The regular neural architecture of the cerebellum inspired different solutions to the long-standing issue of how its circuitry could control motor learning and coordination. Originally, the cerebellar network was modeled using a statistical-topological approach that was later extended by considering the geometrical organization of local microcircuits. However, with the advancement in anatomical and physiological investigations, new discoveries have revealed an unexpected richness of connections, neuronal dynamics and plasticity, calling for a change in modeling strategies, so as to include the multitude of elementary aspects of the network into an integrated and easily updatable computational framework. Recently, biophysically accurate “realistic” models using a bottom-up strategy accounted for both detailed connectivity and neuronal non-linear membrane dynamics. In this perspective review, we will consider the state of the art and discuss how these initial efforts could be further improved. Moreover, we will consider how embodied neurorobotic models including spiking cerebellar networks could help explaining the role and interplay of distributed forms of plasticity. We envisage that realistic modeling, combined with closed-loop simulations, will help to capture the essence of cerebellar computations and could eventually be applied to neurological diseases and neurorobotic control systems.

## Introduction

### The “Realistic” Modeling Approach

In contrast to the classical *top-down* modeling strategies guided by researcher’s intuitions about the *structure-function* relationship of brain circuits, much attention has recently been given to *bottom-up* strategies. In the construction of bottom-up models, the system is first reconstructed through a reverse engineering process integrating available biological features. Then, the models are carefully validated against a complex dataset not used to construct them, and finally their performance is analyzed as they were the real system. The biological precision of these models can be rather high so that they merit the name of *realistic* models. The advantage of realistic models is two-fold. First, there is limited selection of biological details that might be relevant to function (this issue will be important in the *simplification* process considered below). Secondly, with these models it is possible to monitor the impact of microscopic variables on the whole system. A drawback is that some details may be missing, although they can be introduced at a later stage providing proofs on their relevance to circuit functioning (model *upgrading*). Another potential drawback of realistic models is that they may lose insight into the function being modeled. However, this insight can be recovered at a later stage, since realistic models can incorporate sufficient details to generate microcircuit *spatio-temporal dynamics* and explain them on the basis of elementary neuronal and connectivity mechanisms (Brette et al., 2007).

Realistic modeling responds to the general intuition that complexity in biological systems should be exploited rather that rejected (Pellionisz and Szentágothai, 1974; Jaeger et al., 1997; De Schutter, 1999; Fernandez et al., 2007; Bower, 2015). For example, the essential computational aspects of a complex adaptive system may reside in its dynamics rather than just in the structure-function relationship (Arbib et al., 1997, 2008), and require therefore closed-loop testing and the extraction of rules from models running in a virtual environment (see below). Moreover, the multilevel organization of the brain often prevents from finding a simple relationship between elementary properties (e.g., neuronal firing) and higher functions (e.g., motor control or cognition). Network connectivity on different scales exploits local neuronal computations and eventually generates the algorithms subtending brain operations. An important new aspect of the realistic modeling approach is that it is now much more affordable than in the past, when it was less used due to the lack of sufficient biophysical data on one hand and of computational power and infrastructures on the other. Now that these all are becoming available, the realistic modeling approach represents a new exciting opportunity for understanding the inner nature of brain functioning. In a sense, realistic modeling is emerging as one of the most powerful tools in the hands of neuroscientists (Davison, 2012; Gerstner et al., 2012; Markram, 2013). The cerebellum has actually been the work bench for the development of ideas and tools fuelling realistic modeling over almost 40 years (for review see Bhalla et al., 1992; Baldi et al., 1998; Cornelis et al., 2012a; D’Angelo et al., 2013a; Bower, 2015; Sudhakar et al., 2015).

### Cerebellar Microcircuit Modeling: Foundations

In the second half of the 20th century David Marr, in a classical triad, developed theoretical models for the neocortex, the hippocampus and the cerebellum, setting landmarks for the development of theoretical and computational neuroscience (for review see, Ito, 2006; Honda et al., 2013). Since then, the models have advanced alternatively in either one or the other of these brain areas.

The striking anatomical organization of the cerebellar circuit has been the basis for initial models. In 1967, the future Nobel Laureate J.C. Eccles envisaged that the cerebellum could operate as a neuronal “timing” machine (Eccles, 1967). This prediction was soon followed by the theoretical models of Marr and Albus, who proposed the Motor Learning Theory (Marr, 1969; Albus, 1971) emphasizing the cerebellum as a “learning machine” (for a critical vision on this issue, see Llinás, 2011). These latter models integrated a statistical description of circuit connectivity with intuitions about the function the circuit has in behavior (Marr, 1969; Albus, 1971). These models have actually been only partially implemented and simulated as such (Tyrrell and Willshaw, 1992; see below) or transformed into mathematically tractable versions like the adaptive filter model (AFM; Dean and Porrill, 2010, 2011; Porrill et al., 2013).

While Marr himself framed his own efforts to understand brain function by contrasting “bottom up” and “top down” approaches (he believed his approach was “bottom up”), in initial models the level of realism was limited (at that time, little was known on the ionic channels and receptors of the neuronal membrane, by the way). Since then, several models of the cerebellum and cerebellar subcircuits have been developed incorporating realistic details to a different extent (Maex and De Schutter, 1998; Medina et al., 2000; Solinas et al., 2010). In the most recent models, neurons and synapses incorporate Hodgkin-Huxley-style mechanisms and neurotransmission dynamics (Yamada et al., 1989; Tsodyks et al., 1998; D’Angelo et al., 2013a). As far as microcircuit connectivity is concerned, this has been reconstructed by applying combinatorial rules similar to those that have inspired the original Marr’s model. Recently, an effort has allowed the reconstruction and simulation of the neocortical microcolumn (Markram et al., 2015) showing construction rules that may also be used for different brain areas. The approach used for the neocortical microcircuit is based on precise determination of cell densities, on cell morphologies and on a set of rules for synaptic connectivity based on proximity of the neuronal processes (density-morphology-proximity or DMP rule). One question is now whether the construction rules used for the neocortex can also be applied to the cerebellar network. Moreover, since ontogenetic factors play a critical role in network formation, taking a snapshot of the actual state of the mature cerebellar network may not be enough to implement its connectivity and investigate its function. Again, while developmental models have been devised for the cerebral cortex (Zubler et al., 2013; Roberts et al., 2014), their application to the cerebellum remains to be investigated. Therefore, advancement on the neocortical front may now inspire further development in cerebellar modeling.

The most recent realistic computational models of the cerebellum have been built using an extensive amount of information taken from the anatomical and physiological literature and incorporate neuronal and synaptic models capable of responding to arbitrary input patterns and of generating multiple response properties (Maex and De Schutter, 1998; Medina et al., 2000; Santamaria et al., 2002, 2007; Santamaria and Bower, 2005; Solinas et al., 2010; Kennedy et al., 2014). Each neuron model is carefully reconstructed through repeated validation steps at different levels: at present, accurate models of the GrCs, GoCs, UBCs, PCs, DCN neurons and IOs neurons are available (De Schutter and Bower, 1994a,b; D’Angelo et al., 2001; D’Angelo et al., 2016; Nieus et al., 2006, 2014; Solinas et al., 2007a, b; Vervaeke et al., 2010; Luthman et al., 2011; Steuber et al., 2011; De Gruijl et al., 2012; Subramaniyam et al., 2014; Masoli et al., 2015). Clearly, realistic models have the intrinsic capacity to resolve the still poorly understood issue of brain dynamics, an issue critical to understand how the cerebellum operates (for e.g., see Llinás, 2014).

That understanding cerebellar neuron dynamics can bring beyond a pure structure-function relationships was early recognized but the issue is not resolved yet. There are several correlated aspects that, in cascade from macroscopic to microscopic, need to be considered in detail (see below). Eventually, cerebellar functioning may exploit internal dynamics to regulate spike-timing and to store relevant network configurations through distributed plasticity (Ito, 2006; D’Angelo and De Zeeuw, 2009; Gao et al., 2012). The testing of integrated hypotheses of this kind is exactly what a realistic computational model, once properly reconstructed and validated, should be able to promote.

A further crucial consideration is that the cerebellum has a similar microcircuit structure in all its parts, whose functions differentiate over a broad range of sensori-motor and cognitive control functions depending on the specific anatomical connections (Schmahmann and Sherman, 1998; Schmahmann, 2004; Ito, 2006; Schmahmann and Caplan, 2006; D’Angelo and Casali, 2013; Koziol et al., 2014). It appears therefore that the intuition about the network role in learning and behavior of the original models of Marr-Albus-Ito can be implemented now by integrating realistic models into a closed-loop robotic environment. This allows the interaction of the microcircuit with ongoing actions and movements and the subsequent learning and extraction of rules from the analysis of neuronal and synaptic properties under closed-loop testing (Caligiore et al., 2013, 2016). In this article, we are reviewing an extended set of critical data that could impact on realistic modeling and are proposing a framework for cerebellar model development and testing. Since not all the aspects of cerebellar modeling have evolved at similar rate, more emphasis has been given to those that will help more in exemplifying prototypical cases.

### Realistic Modeling Techniques: The Cerebellum as Workbench

Realistic modeling allows reconstruction of neuronal functions through the application of principles derived from membrane biophysics. The membrane and cytoplasmic mechanisms can be integrated in order to explain membrane potential generation and intracellular regulation processes (Koch, 1998; De Schutter, 2000; D’Angelo et al., 2013a). Once validated, neuronal models can be used for reconstructing entire neuronal microcircuits. The basis of realistic neuronal modeling is the membrane equation, in which the first time derivative of potential is related to the conductances generated by ionic channels. These, in turn, are voltage- and time-dependent and are usually represented either through variants of the Hodgkin-Huxley formalism, through Markov chain reaction models, or using stochastic models (Hodgkin and Huxley, 1952; Connor and Stevens, 1971; Hepburn et al., 2012). All these mechanisms can be arranged into a system of ordinary differential equations, which are solved by numerical methods. The model can contain all the ion channel species that are thought to be relevant to explain the function of a given neuron, which can eventually generate all the known firing patterns observed in real cells. In general, this formalism is sufficient to explain the properties of a membrane patch or of a neuron with very simple geometry, so that one such model may collapse all properties into a single equivalent electrical compartment. In most cases, however, the properties of neurons cannot be explained so easily, and multiple compartments (representing soma, dendrites and axon) have to be included thus generating multicompartment models. This strategy requires an extension of the theory based on Rall’s equation for muticompartmental neuronal structures (Rall et al., 1992; Segev and Rall, 1998). Eventually, the ionic channels will be distributed over numerous different compartments communicating one with each other through the cytoplasmic resistance. Up to this point, the models can usually be satisfactorily constrained by biological data on neuronal morphology, ionic channel properties and compartmental distribution. However, the main issue that remains is to appropriately calibrate the maximum ionic conductances of the different ionic channels. To this aim, recent techniques have made use of genetic algorithms that can determine the best data set of multiple conductances through a mutation/selection process (Druckmann et al., 2007, 2008).

As well as membrane excitation, synaptic transmission mechanisms can also be modeled at a comparable level of detail. Differential equations can be used to describe the presynaptic vesicle cycle and the subsequent processes of neurotransmitter diffusion and postsynaptic receptor activation (Tsodyks et al., 1998). This last step consists of neurotransmitter binding to receptors, followed by the opening ion channels or modulation of intracellular cascades, and it is often accounted by stochastic receptor models. The synapses can also be endowed with mechanisms generating various forms of short- and long-term plasticity (Migliore et al., 1995). Appropriate synaptic modeling provides the basis for assembling neuronal circuits.

In all these cases, the cerebellum has provided a work bench that has remarkably contributed to write the history of realistic modeling. Examples are the development of integrated simulation platforms (Bhalla et al., 1992; Bower and Beeman, 2007), the definition of model optimization and evaluation strategies (Baldi et al., 1998; Vanier and Bower, 1999; Cornelis et al., 2012a, b; Bower, 2015), the generation of complex neuron models as exemplified by the Purkinje cells (De Schutter and Bower, 1994a,b; Bower, 2015; Masoli et al., 2015) and the GrCs (D’Angelo et al., 2001; Nieus et al., 2006; Diwakar et al., 2009) and the generation of complex microcircuit models (Maex and De Schutter, 1998; Medina and Mauk, 2000; Solinas et al., 2010). Now, the cerebellar neurons, synapses and network pose new challenges for realistic modeling depending on recent discoveries on neuron and circuit biology and on the possibility of including large-scale realistic circuit models into closed loop robotic simulations.

## Critical Structural Properties of the Cerebellar Network

In the Marr-Albus models, the core hypothesis was that the GCL performs sparse coding of mf information, so that the specific patterns of activity presented to PCs can be optimally learned at the pf-PC synapse under cf control. In these models the cerebellar cortex processes incoming information serially (Altman and Bayer, 1997; Sotelo, 2004) and its output impinges on the DCN, while the IO plays an instructing or teaching role by activating PCs through the cfs. These models reflect the anatomical concept of the cerebellar cortical microzone, which, once connected to the DCN and IO, forms the cerebellar microcomplex (Ito, 1984) representing the functional unit of the cerebellum. Recently, this fundamental modular organization has been extended by including recurrent loops between DCN and GCL and also between the DCN and IO. Moreover, the cerebellum turns out to be divided into longitudinal stripes that intersect the transverse lamella of the folia and can be subdivided into various anatomo-functional regions connected to specific brain structures forming nested and multiple feed-forward and feed-back loops with the spinal cord, brain stem and cerebral cortex. Thus, the cerebellar connectivity, both on the *micro-scale, meso-scale and macro-scale*, is far from being as simple as originally assumed but it rather appears to generate a complex multidimensional hyperspace. A main challenge for future modeling efforts is thus to consider these different scales of complexity and recurrent connectivity.

### Microscale Organization

**Figure 1:**
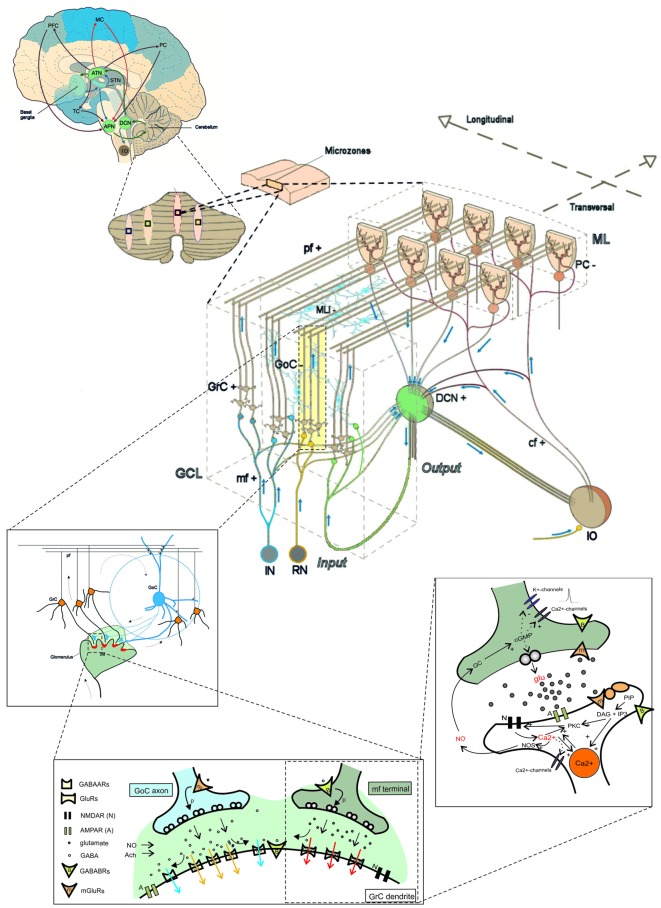
**The multi-level organization of the cerebellum.** This schematic representation shows how the core cerebellar microcircuit is wired inside the whole brain and how it can be further dissected into levels of increasing cellular and molecular complexity. The drawing at the *center* shows the cerebellar cortex subdivided into three layers (GCL, granular cell layer; PCL, Purkinje cell layer; ML, Molecular layer), which contain different types of excitatory and inhibitory neurons (cf, climbing fiber; DCN, deep cerebellar nuclei; GoC, Golgi cell; GrC, granule cell; IO, inferior olive; APN, anterior pontine nucleus; RN, reticular nucleus; MLI, molecular layer interneuron; mf, mossy fiber; pf, parallel fiber; PC, Purkinje cell; the signs indicate the excitatory or inhibitory nature of the cell or fiber). A cortical microzone is connected to IO and DCN to form a cerebellar microcomplex. The expansion to the top, which shows a flattened representation of the cerebellar cortex, indicates how a cerebellar microcomplex can extend to include several microzones located in separated cerebellar regions. A further expansion to the *top* shows the main circuit loops formed by the cerebellum with the cerebral cortex (PFC, prefrontal cortex; MC, motor cortex; PC, parietal cortex; TC, temporal cortex) through the DCN and the anterior thalamic nuclei (ATN) on the efferent pathway and through the anterior pontine nuclei (APN) on the afferent pathway. The connection with basal ganglia (BG) and subthalamic nucleus (STN) is also indicated. The insets to the *bottom* show, expand in cascade the wiring in the granular layer to show glomerular connectivity, glomerular neurotransmission and synaptic transduction mechanisms. The receptors involved (labeled in the inset) and the intracellular cascades include several identified molecular elements (glu, glutamate; PKC, protein kinase C; DAG, diacyl-glycerol; IP3, inositol-triphosphate; PIP, phosphatidyl-inositol-phosphate; NO, nitric oxide synthase; NOS, nitric oxide synthase; NO, nitric oxide; Ca^2+^, calcium ions; GC, guanyl cyclase; cGMP, cyclic GMP; Modified from D’Angelo and Peres, 2011; Mapelli et al., 2014).

**Figure 2:**
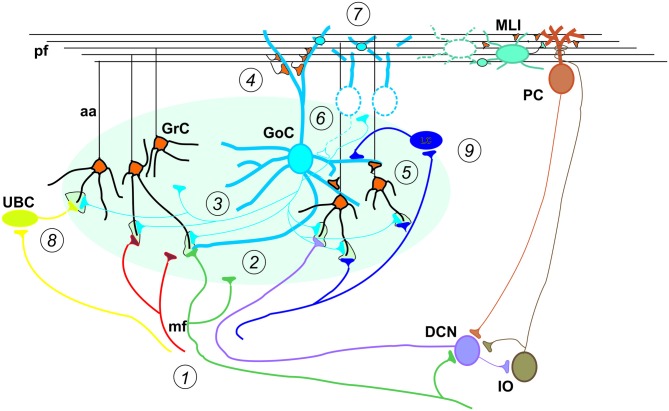
**Special properties of GCL connectivity.** The figure shows schematically the most important properties of GCL connectivity that have emerged from a complex set of physiological and structural experiments. (1) Divergence of mossy fibers onto different cell types. Formation of multiple glomeruli per mossy fiber. Multiple inputs onto the same GrC but different inputs on each granule cell dendrite. (2) Glomerular integration: a cerebellar glomerulus contains a mossy fiber terminal as well as GoC axonal terminals and dendrites. (3) Feed-forward inhibitory loops pass through the MF→GoC→GrC circuit. (4) Feed-back inhibitory loops pass through the MF→GC→GoC→GrC circuit. (5) GrCs activate GoCs both on basal dendrites and apical dendrites (4). (6) GoC→GoC reciprocal inhibition through reciprocal synapses. (7) GoC→GoC communication through gap-junctions. (8) UBC pathway: MF→UBC→ GrC. (9) Lugaro Cell pathway: MF→LC→ GoC. (aa, Ascending axon; other labels and symbols as in Figure 1). Modified from Mapelli et al. (2014).

The cerebellar inputs are elaborated in the GCL before being further processed in the ML and distributed to PCs, from which signals are sent to DCN. While signals flow along the GrC → PC → DCN neuronal chain, they are thought to undergo an initial “expansion recoding” in the GCL followed by a “perceptron-like” sampling in PCs before converging onto the DCN (the validity of these assumptions is further considered below). Local computations in the cerebellar cortex are regulated by two extended inhibitory interneuron networks, one in the GCL and one in the ML. Since the DCN is also activated by mf collaterals, the cerebellar cortex *de facto* operates as a modulator of DCN activity. Finally, the IO → PC → DCN neuronal chain forms another pathway probably implied in controlling network learning and timing capabilities. Recently, relevance has been given to recurrent DCN → GrC and DCN → IO connections, which can directly send output information back to the input. Of great importance for network conceptualization and modeling are not just the convergence/divergence ratios and cell densities reported in Table 1 but also the specific geometries of connectivity reported in Figures 1, 2 (neuron and microcircuit dynamics are considered in the next chapter). It turns out that, differently from the neocortex that has neurons almost isotropically organized inside microcolumns, the cerebellar cortex shows precisely oriented neuronal structures and connections.

**Table 1:** **Statistics of connectivity**.

Source cell	Density	Target cell	Divergence	Convergence	Reference	Species
Glomeruli	3 *10^5^/mm^3^	GrC	1:53	4:1	Solinas et al. (2010)	Rat
		GoC	1:3.6	50:1	Solinas et al. (2010)	
Mf	not known	Glomeruli	not known	not known		Rat
GrC	4 *10^6^/mm^3^	GoC	see aa and pf	see aa and pf	Korbo et al. (1993)	Rat
		PC	see aa and pf	see aa and pf		
GoC	9.000/mm^3^	GrC	1:600	4:1	Korbo et al. (1993)	Rat
Aa	not known	GoC		400:1	Cesana et al. (2013)	Rat
		PC	1:1	*n* (not known):1		
Pf	not known	GoC	1:1.9	1000:1	Walter et al. (2009)	Rat
		PC	1:1	1000:1	Ito (1984)	
		BC/SC	not known	not known		
Cf		PC	1:37 ± 11	1:1	Brown et al. (2012)	Rat
PC	10.100/mm^3^	DCN	1:1	40:1	Korbo and Andersen (1995) and Person and Raman (2012a,b)	Rat
SC	1 *10^5^/mm^3^	PC	1:10~17	7:1	Briatore et al. (2010), Wadleigh and Valenzuela (2012) and Kim et al. (2014)	Mouse
BC	1 *10^5^/mm^3^	PC	1:30	7:1	Briatore et al. (2010), Wadleigh and Valenzuela (2012) and Kim et al. (2014)	Mouse
DCN	50.000–100.000/mm^3^	IO	not known	not known	Baumel et al. (2009)	Mouse
		GoC	not known	not known	Najac and Raman (2015)	
		GrC	1:6	4:1	Ankri et al. (2015)	
					Houck and Person (2015)	
IO	43.900/mm^3^	DCN	1:4	1:1	Schild (1970)	Mouse
					Uusisaari and Knöpfel (2011)	

*The table reports the connectivity between the source and the target cell in the cerebellar circuit, the density of the cerebellar neurons and the divergence/convergence ratios. (Data extracted from Solinas et al., 2010)*.

#### The Double mf and cf Input

The main input to the cerebellum comes through the mfs*.* The mfs originate from neurons located in the brain-stem nuclei (including the cuneate nucleus, vestibular nucleus, reticular nucleus, red nucleus and APN) and spinal cord (dorsal columns). Moreover, relevant to external connectivity, GrCs have recently been shown to receive a blend of modalities from brain-stem and cortical afferences (Huang et al., 2013; Ishikawa et al., 2015). In the GCL, mfs, GrC dendrites, GoC dendrites and axons interact into specialized structures called glomeruli. The mfs emit collaterals forming synapses in the DCN. The other important input originates from a brain-stem nucleus, the IO, giving rise to the cfs contacting PCs and DCNs.

#### The Geometry of Microcircuit Connectivity

The mfs, after entering the GCL, branch longitudinally (i.e., orthogonally to the main axis of the folia) generating numerous “rosettes” (i.e., clusters of glomeruli). The basal GoC dendrites spread around the soma, while the apical dendrites ascend into the ML and the GoC axons remains confined into the GCL also spreading longitudinally (Wu et al., 1999; Sultan, 2001; Sultan and Heck, 2003). There are just 3–5 short GrC dendrites that are connected to as many different glomeruli, whereas the GrC axons pass vertically the PCL and the ML until they divide into pfs running transversally (i.e., along the main axis of the folia). The flattened dendritic trees of PCs form an ordered palisade perpendicular to the folia (Person and Raman, 2012a) and are crossed by pfs connecting arrays of PCs aligned along the pf bundle. The SCs are located in the upper part of the ML and the BCs in the lower of the ML (Briatore et al., 2010; Alcami and Marty, 2013) with the dendritic trees perpendicular to the folium and axons spreading to some distance both along and across the pf bundle. In turn, the cfs branch longitudinally and contact the dendrites of clusters of PCs. Therefore, perhaps the most striking aspect in the cerebellar microcircuit is that, while mfs, cfs, GoC axons and PC dendrites are oriented longitudinally, they are orthogonal to the pfs that cross the PC dendritic trees.

#### The Inhibitory Interneuron Networks

The cerebellum is characterized by two extended inhibitory interneuron networks. The *GCL* *layer inhibitory network* is made of feedforward and feedback loops driven by mfs: (i) the mfs excite GrC and GoC dendrites and these latter inhibit GrCs in a feedforward loop, and (ii) the mfs excite GrCs and then pfs excite GoCs and these latter inhibit GrCs in a feedback loop (Simões de Souza and De Schutter, 2011; Mapelli et al., 2014). The GoCs are interconnected through gap-junctions and reciprocal inhibitory synapses. The *ML inhibitory network* is formed by a series of MLIs (SCs and BCs) activated by pfs and inhibiting PCs in feed-forward (Santamaria et al., 2002, 2007). The MLIs are interconnected through gap-junctions and reciprocal inhibitory synapses (Astori et al., 2009; Alcami and Marty, 2013).

### Mesoscale Organization

Beyond the combinatorial and geometrical architecture described above, which is valid for the whole cerebellar cortex, there are higher orders of organization.

#### Cortical Microzones and Cerebellar Modules

Tracing studies have revealed longitudinal zones that elongate in the rostro-caudal direction and run perpendicular to the long axis of the lobules. The longitudinal zones include the olivocerebellar afferents (cfs) and the corticonuclear (PC) efferents. The somatotopic distribution of cfs are directed to one or two longitudinal zones, while mfs have a more extended transverse branching and terminate in multiple longitudinal zones. Some longitudinal zones can be split into smaller units called microzones. The microzones receiving the same cf inputs from the multizonal microcomplexes and are important for the parallel processing and integration of information coming from mf inputs. Thus, while the neocortex is characterized by microcolums and columns, the cerebellum can be divided into anatomo-functional modules deriving from the assembly of microzones (Cerminara, 2010). Recently, by combining *in vitro* recordings with optogenetics, it has been possible to identify stereotyped patterns of functional synaptic organization between GrCs and PCs, GoCs and MLIs. All these connections displayed position-specific patterns of GrC synaptic inputs that did not strictly match with anatomical boundaries and could connect distant cortical modules, indicating that specific microcircuit connectivity rules have also to be taken into account (Valera et al., 2016).

#### Longitudinal Organization: The Zebrin Stripes

The so-called zones are long cerebellar stripes ranging from the anterior to posterior poles of the cerebellum and can be identified histochemically and functionally (Andersson and Oscarsson, 1978; Apps and Garwicz, 2005; Apps and Hawkes, 2009; Voogd, 2011). Each stripe is defined by the PC type depending on the expression of Aldolase-C (Zebrin II) as well as of other enzymes (e.g., NOS and PKC isoforms) and ionic channels (e.g., TRIP). PCs expressing Zebrin II (Z+) show a slower spontaneous firing (40 Hz) compared to PCs not expressing Zebrin II (Z−; 90–100 Hz; Zhou et al., 2014). Moreover, Z+ and Z− PCs differ as for their ability to generate plasticity at the pf-PC synapse (Wadiche and Jahr, 2005; Wang et al., 2011). It has recently been shown that GoC somata and dendrites are restricted to the same PC Zebrin II stripe (Sillitoe et al., 2008). The restriction of GoCs in specific stripes may influence network activity, since GoCs are connected through gap junctions (Vervaeke et al., 2010) and could have a role in controlling GCL oscillations (Simões de Souza and De Schutter, 2011). The PCs output on specific DCNs is then retransmitted to the IO trough the nucleo-olivary pathway and this pathway has been seen to influence the responses of the IO to their target PCs (Voogd, 2011).

### Macroscale Organization

#### Major Anatomical Subdivisions

The cerebellum, on each side of the midline, is divided into three regions running along the rostral to caudal axis: the vermis, the paravermis and the hemisphere. Each of these regions is folded into lobules and each lobule is subdivided into folia. Remarkably, the afferent and efferent connections of the cerebellar cortex, as well as the corresponding DCNs, are strictly related to this anatomical arrangement, as recently confirmed by viral tracing in experimental animals (Huang et al., 2013; Watson et al., 2014) and MRI data in humans (Balsters et al., 2010; Diedrichsen et al., 2011; Sokolov et al., 2012; Palesi et al., 2015). Projections from the cerebral cortex are conveyed to the anterior pontine nuclei and then relayed mostly to the posterior-lateral parts of the cerebellum through the medium cerebellar peduncle. Projections from the pons and spinal cord are relayed mostly to the vermis and anterior cerebellum through the inferior and superior cerebellar peduncle. These same cerebellar regions project to the spinal cord, brainstem and cerebral cortex through different subdivisions of the DCNs (e.g., see Eccles, 1967; Ito, 1984).

#### Extracerebellar Connectivity and Recurrent Loops

Beyond anatomical details, what is relevant here is that the cerebellum is involved in major connections with brainstem, spinal cord and cerebral cortex as well as with basal ganglia (BG) and hippocampus. These connections generate multiple loops, in which the cerebellum is wired as a pivotal node (Caligiore et al., 2013, 2016; D’Angelo and Casali, 2013).

– The most renowned recurrent loop passes through the IO. The small DCN GABAergic neurons inhibit the IO cells regulating their coupling and oscillations (Najac and Raman, 2015).
– The DCNs are involved in the cerebellar circuitry with a one way connection between the glycinergic DCN, projecting to the GCL, inhibiting GABAergic GoCs and the glutamatergic DCN that excite the GRCs and GOCs (Ankri et al., 2015; Houck and Person, 2015; Gao et al., 2016). A similar connectivity characterizes the medial vestibular nucleus in the vestibulo-cerebellum.
– There are several loops formed with the cerebellum by the brainstem, passing through different cerebellar nuclei (except the dentate) and involving the red nucleus and the reticular nucleus.
– The major loops connecting the cerebellum to the forebrain, start from the dentate nucleus and pass through the anterior ventrolateral thalamus mostly to reach the cerebral cortex, then return through the anterior pontine nuclei and the medial cerebellum peduncle.
– Afferent sensory fibers are relayed to the cerebellum through nuclei located in the spinal cord (e.g., in the Deiter’s columns), brain stem (e.g., the cuneate nucleus), and superior and inferior colliculi.

Functionally, it is important to note that all these loops are normally closed, in that fibers leave and then return to the cerebellum through a different pathway. The most remarkable loops are formed with the cerebral cortex and with the peripheral motor system, so that the cerebellum is actually embedded in loops controlling movement planning and the sensory consequences of movement execution. These loops are the substrates of what are usually referred to as the cerebellar “feed-forward” and “feed-back” controllers (see below).

## Critical Dynamic Properties of the Cerebellar Microcircuit

**Figure 3:**
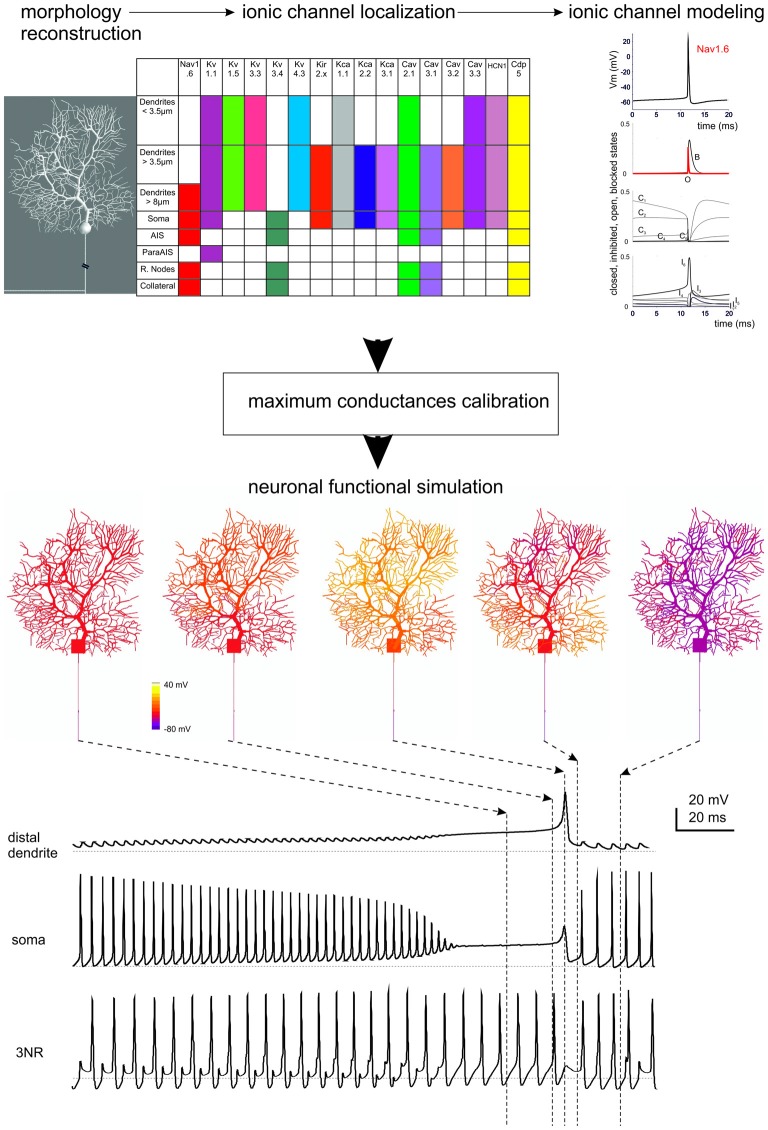
**Ionic channel types, distribution and gating properties in a PC model.** The investigation of cerebellar neurons physiology and biophysics has classically followed the same procedures used for other central neurons. Most experiments have been carried out in mice and rats in acute brain slice preparations with the aim of determining their intrinsic electroresponsiveness. Voltage-clamp analysis of membrane currents has mostly been dedicated to synaptic events, since space-clamp problems have in most cases hindered an accurate determination of current kinetics (except for GrCs, which are electrotonically compact). In some neurons, relevant information has been gained through single-unit and even patch-clamp recordings *in vivo*. Modeling reconstruction has, in most cases, exploited the knowledge of ionic currents identified kinetically and pharmacologically and the corresponding gating models have been derived from ion-channel libraries. The maximum ionic channel conductances have been iteratively adjusted by fitting complex sets of experimental data derived from current-clamp recordings. (Top) The diagram shows a 3D representation of PC morphology. This has been divided into eight distinct sections illustrated in the table on the right and endowed with ionic mechanisms according to immunohistochemical data. The ionic mechanisms include the sodium channel (Nav1.6), LVA and HVA calcium channels (Cav2.1, Cav3.1, Cav3.2, Cav3.3), potassium channels (Kv3.4, Kv1.1, Kv4.3, Kv1.5, Kv3.3), potassium calcium dependent channels (KCa1.1, KCa3.1, KCa2.2), inward rectified potassium channel (Kir2.x), cationic channel (HCN1) and a Ca buffering system composed by Calbindin and Parvalbumin (CDP5). The graph represents the state variables of the Nav1.6 channel during an action potential. C, I, O, B, indicate closed, inactivated, open and blocked states. Vertical dashed lines indicate the approximate action potential threshold (−50 mV). (Bottom) The drawings show PC membrane potential at different times (arrows) during complex bursting (membrane potential is color-coded) in distal dendrites, soma and third node of Ranvier (3NR). At the end of the spike burst, the PC model depolarizes starting from distal dendrites before the depolarization invades the whole dendritic tree. A large Ca spike is the most relevant depolarizing event in terminal dendrites, while fast Na spikes are most evident in AIS. In the 3RN, there is no firing pause during the dendritic Ca spike. (Modified from Masoli et al., 2015).

**Figure 4:**
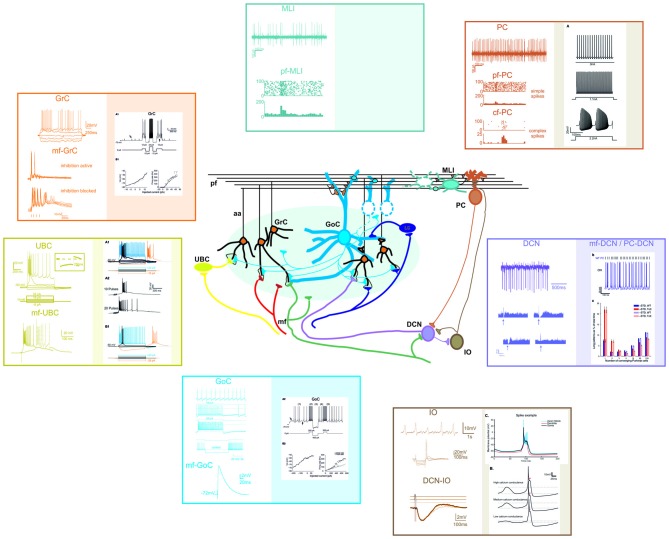
**Different electrophysiological properties of cerebellar neurons and their biophysical modeling.** At present, accurate realistic models have been constructed for most cerebellar neurons, except for MLIs and Lugaro cells. In the different panels, the figure shows schematically the most important properties of cerebellar neurons (left) and their biophysical reconstruction (right). For GCL and IO neurons, example tracings are taken from intracellular current-clamp recordings. For PC, MLI and DCN neurons, example tracings are reported along with raster plots and PSTH of activity. The traces are modified from: (GrC) Experiments: Nieus et al. (2014). Model: Solinas et al. (2010). (UBC) Experiments: Locatelli et al. (2013). Model: Subramaniyam et al. (2014). (GoC) Experiments: Bureau et al. (2000); Forti et al. (2006); D’Angelo et al. (2013b). Model: Solinas et al. (2010). (PC) Experiments: Ramakrishnan et al. (2016). Model: Masoli et al. (2015). (MLI) Experiments: Ramakrishnan et al. (2016). (DCN) Experiments: Rowland and Jaeger (2005); Uusisaari et al. (2007). Model: Luthman et al. (2011). (IO) Experiments: Lampl and Yarom (1997); Lefler et al. (2014). Model: De Gruijl et al. (2012).

The neurons and synapses of cerebellum are amongst the most intensely studied in the whole brain and biophysically detailed models of several cerebellar neurons and synapses are available (Figures 3, 4; Table 2). These models are based on realistic multicompartmental morphologies and incorporate a detailed description of membrane mechanisms including various ionic channels, synaptic receptors, ionic pumps, intracellular calcium dynamics and some cytoplasmic processes. These models, together with detailed connectivity rules, are fundamental to reconstruct realistic microcircuit dynamics.

**Table 2:** **Neuronal electroresponsive properties**.

	Realistic model	Compartments number	Spontaneous frequency	Firing properties	Inward rectification	Resonance frequency
GrC	D’Angelo et al. (2001), Nieus et al. (2006) and Diwakar et al. (2009)	Single Multi	No	Fast spiking, variable presence of adaptation	Fast	~6 Hz
GoC	Solinas et al. (2007a,b) and Vervaeke et al. (2010)	Multi	6 Hz	Fast spiking, adaptation, slow AHP, post-inhibitory rebound	Slow	~6 Hz
UBC	Subramaniyam et al. (2014)	Multi	No	Fast spiking, adaptation, delayed bursting, slow AHP	Slow	–
PC	Masoli et al. (2015)	Multi	40–80 Hz	Fast spiking, adaptation, complex bursting, slow AHP	Slow	–
SC/BC		Multi	20 Hz	Fast spiking, post-inhibitory rebound	Slow	–
DCN	Luthman et al. (2011)	Multi	10–30 Hz	Fast spiking, post-inhibitory rebound	Slow	–
IO	De Gruijl et al. (2012)	Multi	No	Slow spiking, calcium spikes, subthreshold oscillations	Slow	3–10 Hz

*The table reports details about the models available for each type of cerebellar neuron along with a short summary of their characterizing electroresponsive properties*.

### Neuronal Intrinsic Excitability

Neurons of the cerebellum show complex nonlinear properties that are likely to play a key role in controlling network functions. Firstly, several neurons are autorhythmic, with frequencies varying between a few up to around 100 Hz. The spikes have different shapes and properties and can configure various patterns in response to current injection or synaptic activation. Secondly, for some neurons, evidence for resonance in the theta-frequency band has emerged. Thirdly, neurons express non-linear firing properties suitable for processing burst generation and burst-pause responses. Finally, several neurons have inward rectification controlling resting membrane potential and rebound excitation. These properties emerge from the specific ionic channel complement and involve differentially the soma, dendrites and axons. For most of these neurons, there are advanced Hodgkin-Huxley style models, which have helped understanding how the specific electroresponsive properties are generated and as noted above, have set landmarks for realistic modeling strategy (for an extended review see D’Angelo et al., 2016).

*The Purkinje* *cell* is probably the most apparent example of this (for a recent review, see Bower, 2015). Early in the 60’s, Rodolfo Llinas claimed that Purkinje cell dendrites were electrically active (Llinás et al., 1968). Following a lively scientific debate, the demonstration came from a double proof provided by the advent of intracellular PC recordings (Llinás and Sugimori, 1980) followed by the first model of active dendrites (Pellionisz and Szentágothai, 1973, 1974). Then, following precise morpho-electrical reconstruction of a guinea-pig PC (Rapp et al., 1994), the first PC model based on realistic construction principles was presented (De Schutter and Bower, 1994a,b) and then widely used for network simulations for over 20 years (Santamaria et al., 2002; Steuber et al., 2007; Bower, 2010; Maex and Steuber, 2013). Recently, based on the same morphology, a new PC model has been developed using an updated set of ionic channels and accounting for the axonal generation mechanism of simple spikes (Masoli et al., 2015). A compressed version has also been presented (Marasco et al., 2013).

*The granule cell* has been first approximated to a McCulloc-Pitt neuron by a realistic model based on a limited set of ionic currents (Gabbiani et al., 1994). Then GrCs were shown to generate non-linear input-output relationships and were fully modeled based on a more complex set of ionic currents and validated against a rich repertoire of electroresponsive properties including near-threshold oscillations and resonance (D’Angelo et al., 2001). Interestingly, this last model still represents a unique example of full Hodgkin-Huxley style reconstruction based on ionic currents recorded directly from the same neuron, therefore implying minimal assumptions even for the calibration of maximum ionic conductances. The model has subsequently been updated to incorporate detailed synaptic inputs (Nieus et al., 2006, 2014) and to include the dendrites and axon demonstrating the mechanisms of action potential initiation and spike back-propagation (Diwakar et al., 2009). The model has then been used for network simulations (Solinas et al., 2010).

*The DCN cells* have been modeled, although not for all the neuronal subtypes. A model of the glutamatergic DCN neurons, based on realistic morphological reconstruction with active channels (Steuber et al., 2011), was used to analyze synaptic integration and DCN rebound firing after inhibition. More advanced versions have been used to study the dependence of neuronal encoding on short-term synaptic plasticity (Luthman et al., 2011) and the impact of Kv1 channels in spontaneous spike generation (Ovsepian et al., 2013). These models have been used to predict the impact of the cerebellar output on extracerebellar circuits (Kros et al., 2015).

*The IO neurons* were modeled to investigate the interaction of different ionic currents in mono compartmental models (Manor et al., 1997; Torben-Nielsen et al., 2012) showing modifications to sub threshold oscillations (STO) when two neurons where connected through gap junctions. A bi-compartment model (Schweighofer et al., 1999) was able to reproduce the typical STO and the particular spikes generated by the interaction of sodium and calcium currents in the soma/dendritic compartments. A three compartment model was then built to account for the interaction between the dendrites, soma and the AIS in generating the STO and spike output of the IO neurons (De Gruijl et al., 2012). Different versions of IO neuron models have been used to simulate the properties of the IO network (Manor et al., 1997; Torben-Nielsen et al., 2012).

#### Interneurons

The Golgi cells were modeled reproducing the basis of their intrinsic electroreponsiveness, showing complex non linear behaviors such as pacemaking, resonance and phase reset and uncovering the role of gap junctions in oscillatory synchronization (Solinas et al., 2007a,b; Dugué et al., 2009; Vervaeke et al., 2010). The model of UBCs reproduced the nonlinear behaviors of this neuron including bursts, rebounds and the late-onset burst response. This latter property contributes to generate transmission delays in the circuit (Subramaniyam et al., 2014). Concerning MLIs (Llano and Gerschenfeld, 1993; Alcami and Marty, 2013) no detailed conductance-based models are available yet and simplified IF models of these neurons were connected with the PCs to investigate the ML subcircuit (Santamaria et al., 2007; Lennon et al., 2014).

### Synaptic Transmission and Plasticity

A wealth of experimental investigations has addressed the functional properties of cerebellar synapses and will not be considered in detail here (for review see e.g., Mapelli et al., 2014; for the granular layer, Barmack and Yakhnitsa, 2008; for ML). Almost all cerebellar synapses present different forms of short-term plasticity (short-term facilitation: STF; short-term depression: STD) and long-term plasticity (LTP, LTD; De Zeeuw et al., 2011; Gao et al., 2012). In general, short-term plasticity is suitable to regulate transmission during bursts. STD prevails at the mf-GrC synapse, STF prevails at the pf-PC synapse, and STD occurs at the PC-DCN synapses (Häusser and Clark, 1997; Mitchell and Silver, 2000a,b; Nielsen et al., 2004; Sargent et al., 2005; Nieus et al., 2006; DiGregorio et al., 2007; Szapiro and Barbour, 2007; Kanichay and Silver, 2008; Duguid et al., 2012; Powell et al., 2015; Wilms and Häusser, 2015; van Welie et al., 2016). While neurotransmitter dynamics involving vesicular release as well as postsynaptic receptor desensitization proved critical for controlling neurotransmission dynamics, an intriguing observation has been that spillover in the cerebellar glomerulus and in the ML might have a more important role than expected (e.g., see Mitchell and Silver, 2000a,b; Szapiro and Barbour, 2007).

Likewise, there are more than 15 forms of long-term synaptic plasticity in the cerebellar network, appearing both as LTP or LTD with multiple and different mechanisms of induction and expression (for review, see Ito, 2002; Gao et al., 2012; D’Angelo, 2014). Plasticity has been reported not just in acute brain slices but also *in vivo* (Jörntell and Ekerot, 2002; Roggeri et al., 2008; Diwakar et al., 2011; Johansson et al., 2014; Ramakrishnan et al., 2016), revealing that patterned sensory inputs can determine a complex set of changes encompassing multiple synaptic relays. Importantly several of the cerebellar synapses may show forms of spike-timing-dependent plasticity (STDP), linking intracerebellar oscillations to the ability of generating plasticity (D’Angelo et al., 2015; Garrido et al., 2016; Luque et al., 2016). Understanding the importance of these forms of plasticity may greatly benefit from integrated network modeling. At present, models incorporating dynamics presynaptic vesicle cycling (Tsodyks et al., 1998) have been developed for the mf-GrC, mf-GoC, GoC-GrC and GrC-GoC synapses (Nieus et al., 2006, 2014).

### Microcircuit Dynamics: Timing and Learning

The cerebellar microcircuit has been shown to develop dynamic behaviors, although their investigation is still limited. The EEG cannot normally be recorded from the cerebellum, although some MEG data have been reported showing increased power in the theta-band during motor processing (Gross et al., 2001, 2002). Recordings in the experimental animal *in vivo* have focused on PC discharge patterns. PCs have been shown to activate in spots forming transient clusters (Velarde et al., 2004), to exploit burst-pause coding (Herzfeld et al., 2015) and to encode the prediction of ongoing motor states (Balsters et al., 2010). A recent report has shown that locomotion was associated with widespread increased activity in GrCs and interneurons, consistent with an increase in mossy fiber drive, and that dendrites of different PC showed increased co-activation, reflecting increased synchrony of climbing fiber activity. At the same time, responses to external stimuli in all three cell types were strongly suppressed showing that climbing and mossy fiber representations can shift together within a fraction of a second between responses to movement-associated or external stimuli (Ozden et al., 2012). However, the spatio-temporal reconfiguration of signals expected to occur in the GCL remains to be fully addressed *in vivo* and it is not fully clear how signals coming from different sources are redistributed through the different internal channels of the cerebellum.

Relevant to cerebellar circuit dynamics are its oscillating and resonant properties. On one hand, the GCL can be entrained into coherent oscillations by external inputs, possibly exploiting the resonance properties of its neurons (Pellerin and Lamarre, 1997; Hartmann and Bower, 1998; D’Angelo et al., 2001; Courtemanche et al., 2002, 2013; Solinas et al., 2007a; D’Angelo and De Zeeuw, 2009; Gandolfi et al., 2013; Garrido et al., 2016). On the other hand, spontaneous oscillations occur in the IO, that might have the role of coordinating cerebellar activity generating patterns that could be used for timing motor, sensory and cognitive tasks (Lampl and Yarom, 1997; Jacobson et al., 2008; Llinás, 2014). In 2011, these two observations have been merged with a large set of experimental data to propose a 3-level hypothesis, in which: (1) the spatio-temporal reconfiguration of incoming signals in the GCL is followed by; (2) their synthesis in the ML and DCN; while (3) the DCN/PC/IO loop controls a modular synchronization of cerebellar sub-fields based on circuit recurrent dynamics and selective frequency-dependent signal transmission (D’Angelo, 2011). The issue of oscillations is particularly relevant not just for microcircuit computation but also for microcircuit learning through STDP rules (see also “Model Simplification and Implementation in Closed-loop Robotic Testing” Section below). Once again, *timing* to *learning* appear as complementary aspects of the same mechanisms rather than alternative mechanisms of function, as it was suggested by the original models (Marr, 1969; Eccles, 1973).

### Signal Transmission in Local Microcircuits

Despite its extensive investigation, several fundamental issues about signal transmission in local microcircuits are still incompletely understood.

There has been a long debate, which is not fully resolved yet, on the modality of PC activation by GCL inputs. While punctuate peripheral stimulation *in vivo* generates activity spots on the cerebellar surface (Bower and Woolston, 1983; Rokni et al., 2007), local pf stimulation elicits stripes of activity along the pf bundles (Ebner and Pasalar, 2008; Ebner, 2013). A recent work using localized Glu uncaging in acute cerebellar slices suggests that the organization of connections between the GCL and PCs may actually be even more complex than originally thought (Valera et al., 2016). From a functional viewpoint, following GCL stimulation, high-frequency modulated bursts are reliably transmitted vertically from the GCl to PCs, while only low-frequencies are transmitted transversally along the pfs (Mapelli et al., 2010). This observation suggested that a frequency-dependent selection of transmission lines, together with a specific micro-connectivity, may allow the formation of functional modules of active spots emerging vertically at the intersection of multiple pf bundles running along the folia with cfs fibers branching orthogonally to them (D’Angelo, 2011). At these intersection points, PCs may be able to decode the phase of IO oscillations and regulate pf gain (Ohtsuki et al., 2009).

A correlated issue concerns signal spread in the ML and PC inhibition. The pure feed-forward inhibition of PCs has inspired initial functional models taking the move from the observation that SCs and BCs inhibit PC activity with specific spatial organization and timing along and across the pf bundle (Eccles, 1967; Ito, 1984). This structural-functional relationship has recently been revisited highlighting the differential effect of inhibition on PC excitation mediated by aa and parallel fiber synapse (Mann-Metzer and Yarom, 1999, 2000, 2002; Santamaria et al., 2002, 2007; Mittmann et al., 2005; Santamaria and Bower, 2005; Mittmann and Häusser, 2007; Rieubland et al., 2014). Several dynamic phenomena have been reported to intervene in determining how the ML actually operates. SCs are pacemaking and are electrically coupled thus forming an oscillating interneuron network (Mann-Metzer and Yarom, 1999, 2000, 2002; Alcami and Marty, 2013). The analysis of these electrical and chemical SC microcircuits has recently revealed that transitivity of chemical connectivity is directed vertically in the sagittal plane, and electrical synapses appear strictly confined to the sagittal plane (Rieubland et al., 2014). The effect of ML inhibition is not confined to regulate PC activity, but it can also regulate generation of LTD and LTP at pf-PC synapses (Mittmann et al., 2005; Mittmann and Häusser, 2007). On the side of ML coding, SC inhibition deeply affects the burst-pause pattern of PC output (Steuber et al., 2007; Herzfeld et al., 2015). Moreover, a form of interconnectivity between PCs has been proposed to generate traveling waves of activity in the ML (Watt et al., 2009).

Finally, the dynamics of the IO-PC-DCN subcircuit remain still incompletely understood. The well-known contention about the role of cfs, that has been proposed either to control cerebellar learning or timing (Ito, 2000; Jacobson et al., 2008; Llinás, 2009, 2011, 2014), is not yet over. What is becoming clear is that this subcircuit has all the ingredients to subserve both functions. The IO operates as a pattern generator exploiting gap-junctions and local synaptic inhibition coming from the DCN in order to organize internal activity patterns that are then conveyed to PCs (Jacobson et al., 2008; Chen et al., 2010; Libster et al., 2010; Lefler et al., 2013; Libster and Yarom, 2013). This cf pattern, in turn, could be used to select mossy fiber patterns in specific groups of PCs. It can be argued that the coincidence of these cf and mf patterns could be instrumental to generate various forms of plasticity at PC and DCN synapses (see D’Angelo, 2014) raising again the duality of the timing-plasticity issue in the cerebellar circuit.

## Realistic Models of the Cerebellar Microcircuit

Realistic models of the cerebellar network have to take into account a series of experimental observations, some used for construction, others for validation. In general, morphological measurements are the most relevant for constructing the network structure, electrophysiological data are needed to implement neurons and synaptic models, microcircuit-scale functional measurements (imaging and electrophysiology) are fundamental for validation.

### The Most Compelling Example: The Model of the GCL Subcircuit

#### Construction

**Figure 5:**
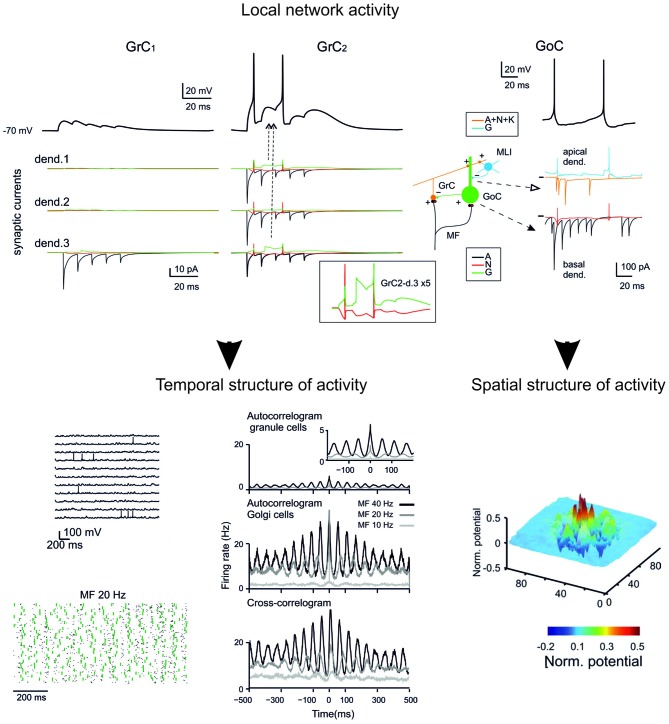
**GCL modeling.** The reconstruction of the microcircuit model of the GCL involves a precise representation of neurons, synapses and network connectivity. Interestingly, the model accounted for all the spatio-temporal dynamics of the GCL known at the moment. The model can therefore provide relevant information about the inner structure of neuronal activity during specific patterns of activity and reveal the relationship between individual synaptic and neuronal elements and the ensemble network response. (Top) synaptic currents in the dendrites of two different GrCs and receptor-specific components (AMPA, A; NMDA, N; GABA, G). (Bottom) Spatio-temporal dynamics of the network under noisy inputs reveal coherent low-frequency oscillations in the GC populations (left). Spatial response of GCs to a collimated mf bursts reveal a center-surround structure (right). (Modified from Solinas et al., 2010).

The wealth of anatomical data reported above (Figures 1, 2) and of cellular data (Figures 3, 4) provides the basis for reconstructing the cerebellar microcircuit (Figure 5). The state of the art for the cerebellar GCL is currently set by the 2010 model (Solinas et al., 2010), which was intended to generate a core computational element of the GCL microcircuit (about 10,000 neurons). This model was built by carefully reproducing the cerebellar GCL network anatomical properties and then validating the response against a large set of available physiological data. A peculiarity of the cerebellar network is that of being highly defined in terms of number of elements, convergence/divergence ratios and even in the number of synapses impinging on individual neurons. Moreover, the geometric orientation of processes is not isotropic but rather geometrically oriented, so that this network is quasi-crystalline in nature. This has allowed the application of a “direct approach”, in which:

– The appropriate number of neuronal elements has been randomly dislocated in a 3D space (density).
– The connectivity rules have been implemented to respect the convergence/divergence ratios.
– The connections have been limited to specific network sub-spaces with well defined innervation territories. This, together with the estimates of cell densities and of the number of synapses, allowed to implement an equivalent 3D connectivity even if the axonal plexus was not represented explicitly.
– The neurons, though very accurate, had an equivalent rather than a realistic morphology, either monocompartmental (GrCs) or multicompartmental (GoCs).

Given that the data were sufficient to determine microcircuit connectivity, it was not necessary to implement DMP rules (see below). Moreover, since the neurons were very accurate in reproducing the neuronal electrophysiological properties (Table 2), there was no need to implement realistic morphologies. Therefore, this network represents a “special case” of a more general network reconstruction procedure, as explained below.

#### Validation

Network validation has been performed against a relevant experimental dataset:

– First of all, it was considered whether the model neurons, which were calibrated beforehand on acute slice data (D’Angelo et al., 2001; Nieus et al., 2006; Solinas et al., 2007a,b), showed properties observed using patch-clamp recordings *in vivo* (Rancz et al., 2007; Arenz et al., 2008; Duguid et al., 2012, 2015; Chadderton et al., 2014). This actually happened, suggesting that a simulation of the role played by specific ionic channels during network processing is actually possible.
– Secondly, it was assessed how the model network reacted to random inputs distributed across the mfs. The model correctly generated coherent GrC oscillations in the theta band (Pellerin and Lamarre, 1997; Hartmann and Bower, 1998) provided that an appropriate balance between the MF and PF input to GoC was maintained.
– Thirdly, it was considered whether the high-pass filtering properties of the GCL emerged. Again this happened, with a correct cut-off around 50 Hz. Importantly, this property depended on NMDA receptors but much less so on GABA-A receptors, as observed experimentally (Mapelli et al., 2010).
– Finally, the network response to collimated mf bursts was tested. According to previous observations using MEA recordings, the typical center-surround organization of GCL responses emerged (Mapelli and D’Angelo, 2007).

Therefore, the GCL network model successfully reproduced the whole set of functional properties known at that time, suggesting that it could be used for predicting emerging network behaviors. Nonetheless, several issues remained unresolved, mostly concerning the GoC inhibitory network, and the range of network properties has in the meantime been extended by new findings.

– The relative weight of the feed-forward and feed-back inhibitory loop generated by GoCs was a free parameter, whose impact was explored explicitly. A strong feed-back loop favored coherent GCL network oscillations, as predicted by a previous modeling layout (Maex and De Schutter, 1998), while a strong fed-forward loop was needed to implement the time-windowing effect (D’Angelo and De Zeeuw, 2009). It still remains unclear how the two loops balanced. It is possible that the oscillating mode dominates over large network areas and that selective mf inputs to GoCs project restricted regions into the time-window mode, a hypothesis that needs to be tested (Duguid et al., 2015).
– The inhibitory input to GoCs was supposed to derive from MLIs, but now this hypothesis is less creditable, since recent data support the existence of inhibitory GoC-GoC connections (Hull and Regehr, 2012).
– The excitatory input to GoCs is more complex than previously thought, GrCs form contacts onto GoC dendrites (Cesana et al., 2013), and GoCs are connected through gap-junctions (Dugué et al., 2009; Vervaeke et al., 2010).
– The modality of GoC-GrC connectivity in the glomerulus is not clear yet. While each GrC receives a single inhibitory contact from GoCs, it is not clear if all the GrCs in each individual glomerulus receive inhibition from the same GoC axon or rather if they receive connections from different GoCs.
– Finally, the small-scale of the 2010 network precluded the analysis of extended spatio-temporal effects, for example of those concerning interaction of different active clusters and the spatial distribution of responses along the pf axis.
– The microscopic structure of GCL network activation can now be compared with the multispot two-photon microscopy data, which provide a new level of microcircuit validation (Gandolfi et al., 2014).

Eventually, improvements of specific structural properties and of membrane and intracellular mechanisms could also be considered. For example, the dendrites of GoCs are likely to be active and this has to be accounted for in future models (Rudolph et al., 2015). Multicompartment GrC models perform better than monocompartment ones in controlling spike properties and delays (Diwakar et al., 2009) and so they should be developed and adopted for all neurons in the network model. Specific issues concern the cerebellar glomerulus: at present, this structure has a fictive morphology but it could be designed to incorporate a closed diffusion space allowing the generation of glomerular homeostatic mechanisms balancing excitatory and inhibitory neurotransmitter release during repetitive synaptic activity (Mapelli et al., 2014; Nieus et al., 2014). Another specific issue concerns the mechanisms of postsynaptic calcium regulation, signal transduction and plasticity in GrCs and GoCs dendrites, for reason that will become evident below.

The model of the GCL is fundamental since it generates the input to the subsequent stages of the cerebellar cortex. Although, in a local perspective, a microcircuit made of GrCs and GoCs is enough to generate meaningful outputs for ML and PCs, the incorporation of the GCL in an extended macrocircuit requires a set of extensions. These concern additional control subcircuits that include the UBC subcircuit, that predicted to play an important role in generating delay lines inside the GCL (Kennedy et al., 2014), and the LC subcircuit, that provides a control loop regulating GoC activity (Dieudonné and Dumoulin, 2000; Barmack and Yakhnitsa, 2008).

### Perspectives for Modeling Other Cerebellar Network Subcircuits and The Whole Cerebellar Network

The GCL network provides the most advanced computational model of the cerebellum at the moment. The impact of GCL modeling becomes even more relevant once the GCL output is used to activate the ML. At this level, mapping of GCL activity onto PCs and MLIs occurs serially, as there is no evidence of direct feed-back from the ML to the GCL (though it occurs through DCN and extracerebellar loops, see also below). A reference model for the ML has been proposed over 10 years ago to explain PC activation (Santamaria et al., 2007), but the main connectivity aspects of BCs and SCs with PCs need now to updated with recent data that revealed potentially important physiological and molecular details. For example, ephaptic synapses need to be added on the PC axonal initial segment (Blot and Barbour, 2014) and short-term plasticity needs to be implemented at all the ML synapses (Liu et al., 2008; Lennon et al., 2015). Likewise, while models for the fundamental properties of IO and DCN neurons are available, they also need to be updated. For example, IO neuron axonal burst generation (Mathy et al., 2009) still needs to be resolved. All these properties are likely to have a relevant impact on cerebellar computation dynamics. The same connectivity inside the IO-DCN-PC subcircuit has never been modeled in full although relevant progress has been done (De Schutter and Steuber, 2009; Steuber and Jaeger, 2013). In principle, the IO-DCN-PC subcircuit should be modeled independently and tested and then wired with the cerebellar cortical model.

A first series of effects is expected from the integration of the different subcircuits (granular, molecular and IO-DCN-PC) into a whole-cerebellum network model. This assembly, by including a set of recurrent loops, breaks down the serial processing scheme adopted when modeling the cerebellar subcircuits separately. In this way, the intrinsic dynamics of the IO-DCN-PC subsystem will be integrated with the activity patterns carried by the mfs and processed in the GCL and ML. Eventually, this whole-cerebellum network model will help facing the basic question of how PC and DCN firing is regulated by the cerebellar cortical circuit activity.

A second series of effects is expected from the integration of the whole-cerebellum network model into extracerebellar loops. This step is essential to analyze how the cerebellar network operates. For example, properties like resonance or STDP are relevant only in the context of rhythmic patterns of activity in closed-loop circuits formed by the cerebellum with the DCN (Kistler and De Zeeuw, 2003), the cerebral cortex, brain stem and spinal-cord. The needing of connecting the cerebellum model with external brain structures brings about a series of additional modeling questions.

#### Relevant Properties of the mf Input

Several anatomical and functional observations become relevant when considering the internal and external connectivity of the cerebellum. The mfs connecting to a certain GrC are probably not all of the same nature but rather they come from different sources. For example, there are GrCs receiving combinations of cortical and spinal afferences and some show a multimodal response to sensory stimulation (Huang et al., 2013; Ishikawa et al., 2015). Thus, each GrC may work as a coincidence detector of different signal sources. However, in some areas GrCs may operate as threshold detectors for the intensity of signal sources deriving from a specific modality or somatic subregions (Bengtsson and Jörntell, 2009). Implementing these connections requires to know how mfs from different sources combine in individual GrC and requires therefore a specific redistribution of glomeruli inside the GCL (Billings et al., 2014). Ideally, the combination of different fibers in GrCs allows direct coincidence detection of signals from different areas carrying “congruent” information that needs to be associated before further processing in the cerebellum. Some mfs also come from the DCN imposing further constraints on the internal distribution of connections. The GrCs receiving the internal feed-back from DCN may be able to associate the coincidence between DCN and extracerebellar inputs. These observations suggest that understanding the cerebellar GCL should consider the distribution of glomeruli deriving from mfs originating from various sources.

#### Relevant Properties of Zonal and Regional Organization

Perhaps the aspect most relevant to cerebellar modeling on the mesoscale is the organization of subcircuits, in which the cfs and the mfs contacting a certain group of PCs and DCN neurons are connected to the same area of origin to form fully connected cerebellar modules. Furthermore, the cerebellar modules can be organized according to the longitudinal stripes, in which some neuronal and synaptic mechanisms are differentiated depending on the type (Z+ or Z−) of the stripe (Wadiche and Jahr, 2005; Wang et al., 2011; Zhou et al., 2014). In turn, a model on the macroscale has to be composed of multiple modules, each one connected to specific extracerebellar regions. These aspects will have to be considered once the cerebellum model will be wired with extracerebellar areas (see below).

## New Modeling Strategies for New Challenging Questions

Realistic cerebellar modeling has to face two main challenges. First, it has to able to incorporate realistic morphologies and to improve details on the molecular and cellular microscale. Secondly, it has to be expanded toward the mesoscale and macroscale. In order to do so, a general and flexible implementation strategy is needed, and in this process cerebellar modeling has once again been acting to promoting the development of general model strategies (Bhalla et al., 1992; Bower and Beeman, 2007).

The cerebellar network is probably the most ordered structure of the brain, and this has allowed a precise modeling reconstruction of its internal connectivity based on extended datasets derived from mice and rats (Maex and De Schutter, 1998; Medina and Mauk, 2000; Medina et al., 2000; Solinas et al., 2010). A further advancement would benefit of an approach based on structured multiscale simulators (Hines and Carnevale, 2001; Bower and Beeman, 2003; Gleeson et al., 2007; Ramaswamy et al., 2015). This would allow to extend cerebellum modeling performed in mice and rats to other species (e.g., humans) and to paracerebellar structures, including the dorsal cochlear nucleus in all vertebrates and the paracerebellar organs in electric fishes (Oertel and Young, 2004; Requarth and Sawtell, 2011; Kennedy et al., 2014). This approach would facilitate the incorporation of new cell types (like the UBCs or the LCs), provided that their detailed single neuron models are available. This approach can host morphological and functional variants of the different neurons, thus moving from canonical neuronal models to neuron model families expressing all the richness of electrophysiological properties that characterize biological networks.

The cerebellum is fundamentally a plastic structure and its function is hard to understand if plasticity is not considered. The cerebellum drives adaptation through plasticity. Moreover, the cerebellum attains the adult network organization through a blend of plastic processes guided by the interaction of genetic programs with epigenetic cues. Thus the interaction of the cerebellar network with the rest of the brain and with ongoing behavior is key not just to determine how the cerebellum operates but also how the cerebellum forms its internal structure and connections. Plasticity during development and in adulthood are probably the most fascinating aspects of the cerebellum and pose challenging questions for modeling.

In adulthood, the cerebellar synapses express various forms of plasticity with learning rules showing different pattern sensitivity, induction and expression mechanisms (D’Angelo, 2014). The corresponding learning rules are embedded into these mechanisms and although it would be desirable that these are eventually represented using dynamics synaptic models (Migliore et al., 1995, 1997, 2015; Tsodyks et al., 1998; Migliore and Lansky, 1999; Rothman and Silver, 2014) at present no such models are available. Nonetheless, theoretical rules based on Hebbian coincidence detectors and STDP have been developed in some cases (Garrido et al., 2016; see below). Eventually a realistic model incorporating learning rules resolved at the molecular level should be able to give insight on the adaptable properties of the network.

As far as ontogenetic network self-organization is concerned, a reference model has been developed for the cerebral cortex accounting for synapse formation through an interaction/pruning process guided by Hebbian rules (Zubler et al., 2013). The dendrite extension/pruning process would by itself solve problems like the crystalline convergence/divergence ratio of the mf-GrC relay and of the cf-PC connectivity. In a way, it can be envisaged that the selection rules of DMP algorithm will eventually be implemented using growing plastic rules. Moreover, once connection pathways are prescribed, the self-organizing system should be able to generate the appropriate distribution of the mf-glomeruli into the cerebellar GCL and to prime the ontogenetic development of the whole network, aligning transmission channels and optimizing circuit performance by setting the appropriate associations of fiber types.

Thus the problem is not just to determine and model the plasticity rules, but also to apply them to the network, as this would require the cerebellum model to be inserted in a whole-brain system interacting with the environment.

## Model Simplification and Implementation in Closed-Loop Robotic Testing

The ultimate challenge appears then to run the whole-cerebellum network model in a simulated brain operating in closed-loop. While a radical approach is out of reach at the moment (it would require, in addition to fully developed cerebellum models, also realistic models of large brain sections outside the cerebellum), a first attempt has been done by reducing the complexity of cerebellar models and using simplified versions to run closed-loop robotic simulations (Casellato et al., 2012, 2014, 2015; Garrido et al., 2013; Luque et al., 2014, 2016).

### Complexity Reduction

The way complexity reduction is achieved is critical, since it has to be performed in a way that preserves the fundamental biological properties relevant to the process under investigation. Two recent approaches have been proposed. Realistic PC models currently involve about 1500 electrical compartments and up to 15 active ionic conductances (De Schutter and Bower, 1994a,b). This complexity has been remarkably reduced by applying Strahler’s analysis to reduce up to 200-fold the run time but yet maintaining an appropriate response to synaptic inputs (Marasco et al., 2012, 2013). Likewise, the granular layer network has been simplified using analytical tools by increasing the simulation speed at least 270 times but yet reproducing salient features of neural network dynamics such as local microcircuit synchronization, traveling waves, center-surround, and time-windowing (Cattani et al., 2016). In all these cases, a well defined relationship is maintained between the simplified models and their more complex realistic counterparts. These attempts open the way to a guided simplification procedure, at least for some cerebellar neurons and subnetworks. When the whole cerebellar network has to be represented in a macro-scale model, simplifications that are computationally efficient may be preferable in a first instance. Clearly, in this case a *top-down* approach is adopted and the relationship of the simplified model with the real system is a matter of speculation. This approach has been used to generate cerebellar spiking networks (SNN) allowing to reproduce a single basic cerebellar module running with high efficiency in a robotic controller yet maintaining some fundamental features of neurons and connections (Casellato et al., 2012, 2014, 2015; Garrido et al., 2013; Luque et al., 2014, 2016). For example, in these models, neurons were represented by integrate-and-fire single-compartment elements, the local inhibitory interneuron networks were not included and the GCL was not fully implemented resorting to the concept of a non-recurrent states in a liquid-state machine (Yamazaki and Tanaka, 2007). Nonetheless, the model incorporated multiple forms of bidirectional plasticity at the PC and DCN synapses. This compromise had to be accepted in order to generate a spiking cerebellum model running in *real-time* inside a closed-loop robotic control system and to perform system level analysis of complex tasks like active manipulation.

### Spiking Neural Networks of the Cerebellum

**Figure 6:**
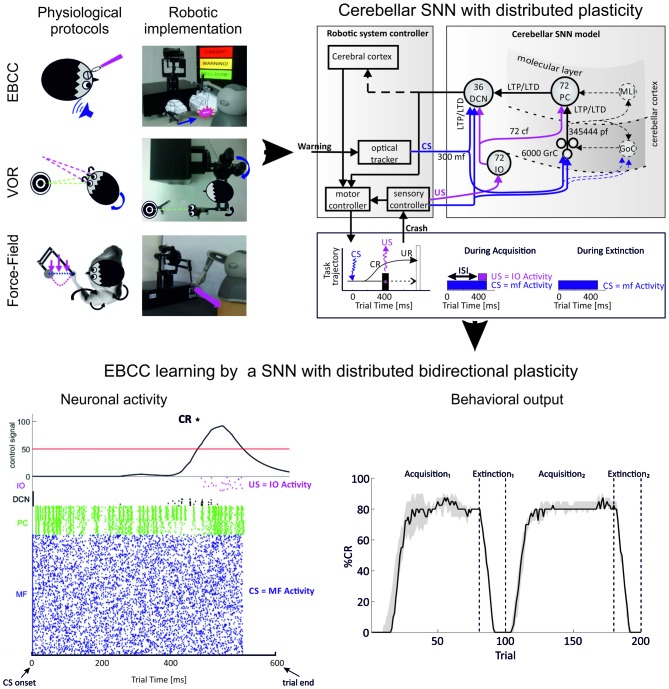
**Simulating an associative learning task using a cerebellar spiking neural network (SNN).** The cerebellum circuit was simplified and embedded into a robotic control system, in which it provided the substrate to integrate spatio-temporal information in different associative learning tasks. *Real robot paradigms (top left panel)*: eye blink classical conditioning (EBCC)-like, vestibulo-ocular reflex (VOR) and upper limb reaching perturbed by force fields. The EBCC-like Pavlovian task is reproduced into the robotic platform as a collision-avoidance task. The conditioned stimulus (CS) onset is based on the distance between the moving robot end-effector and the fixed obstacle placed along the trajectory, detected by the optical tracker. The unconditioned stimulus (US) is the collision event. The DCNs trigger the conditioned response (anticipated stop). The VOR is reproduced into the robotic platform by using the second joint of the robotic arm as the head (imposed rotation) and the third joint (determining the orientation of the second link) as the eye. The misalignment between the gaze direction and the environmental target to be looked at is computed through geometric equations from the optical tracker recording. The DCNs modulate the eye compensatory motion. The perturbed reaching is reproduced into the robotic platform by applying a viscous force field on the moving robotic arm by means of the other robotic device attached at its end-effector. The DCNs modulate the anticipatory corrective torque. (Modified from Casellato et al., 2014). *EBCC-like control system embedding spiking cerebellar network (top right panel).* US is fed into the cf pathway; CS into the mf pathway. CS and US co-terminate (as in the “delay” EBCC). The SNN learns to produce conditioned responses (CRs), i.e., a stop of the robotic arm (collision avoidance) anticipating the US onset. The figure highlights the major forms of plasticity embedded in the cerebellar network and driving the learning, namely synaptic long-term potentiation (LTP) and synaptic long-term depression (LTD), both at cortical and nuclear levels (distributed plasticity). The protocol is made up of acquisition and extinction phases; in the acquisition trials CS-US pairs are presented at a constant Inter-Stimuli Interval (ISI); in the extinction trials CS alone is presented. Each trial lasts 600 ms. The number of cell in the circuit is indicated. All labels as in previous figures. (Modified from D’Angelo et al., 2015). *Network activity and output behavior during EBCC training (bottom panel).* After learning, the response of PCs to inputs decreases, and this increases the discharge in DCN neurons (raster plot and integral of neuronal activity, left). Since the DCN spike pattern changes occur before the US arrival, the DCN discharge accurately predicts the US and therefore facilitates the release of an anticipatory behavioral response. Number of CRs (%) along trials (80 acquisition trials and 20 extinction trials for two sessions in a row; CR% is computed as percentage number of CR occurrence within blocks of 10 trials each). The black curve (right plot) represents the behavior generated by the cerebellar SNN equipped with only one plasticity site at the cortical layer (median on 15 tests with interquartile intervals). Despite uncertainty and variability introduced by the direct interaction with a real environment, the SNN progressively learns to generate CRs anticipating the US, to rapidly extinguish them and to consolidate the learnt association to be exploited in the re-test session. (Modified from Casellato et al., 2015; D’Angelo et al., 2015; Antonietti et al., 2016).

Despite the simplicity of the cerebellar SNN (Figure 6), the robots that incorporated it revealed remarkable emerging properties (Casellato et al., 2012, 2014, 2015). The SNN robots correctly performed multiple associative learning and correction tasks, which ranged from eye-blink conditioning to vestibulo-ocular reflex (VOR) and force-field correction. Importantly, the robots were not designed for any specific one of these tasks but could cope equally well with all of them demonstrating generalized learning and computational capabilities. The robots could also generalize their previous stored patterns to analogous cases with a learning rate approaching that observed in real life. This system could easily fit human EBCC data predicting dual-rate learning in the network. Again, the outcome of the closed-loop simulation have been validated against real experiments carried out in humans (Monaco et al., 2014; D’Angelo et al., 2015) and the challenge is now to see whether it is predictive with respect to human pathologies.

An important aspect of these models is to incorporate *learning rules* that allow to test the impact of learning on cerebellar computation. While a precise correspondence with long-term synaptic plasticity is not at the level of molecular mechanisms (we are dealing with simplified models by the way), these learning rules can effectively capture the learning dynamics of the system. Importantly, faster learning rates at PC than DCN synapses allow fast acquisition and subsequent transfer of memory in a consolidated state (Luque et al., 2014) and STDP rules allow learning to accurately match the network temporal dynamics (Luque et al., 2016). These models allowed to evaluate the impact of known forms of bidirectional LTP/LTD at pf-PC, PC-DCN and mf-DCN synapses and to predict a critical role for plasticity at IO-DCN synapses. The implementation of GCL plasticity poses a formidable problem as it is hard to determine its supervision process. A recent proposal suggests that the issue could be solved by exploiting multi-step learning with an initial pattern storage in the inhibitory interneuron network formed by Golgi cells (Garrido et al., 2016).

### Advanced Robotic Simulations of Manipulation Tasks

**Figure 7:**
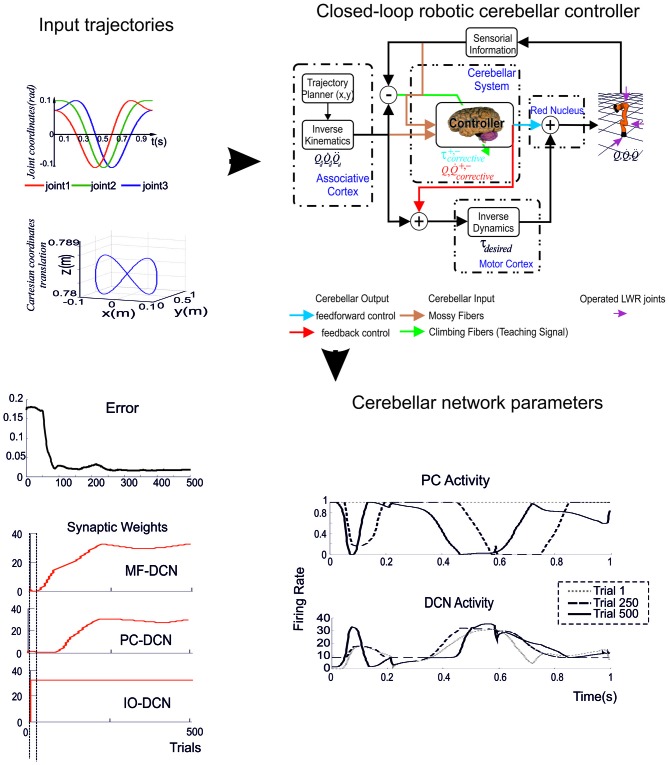
**Biologically plausible cerebellar control loops.** (Top left) The target trajectory followed by the robotic arm model is defined along three degrees of freedom in joint coordinates and Cartesian coordinates. (Top right) In the feedback cerebellar (recurrent) control loop, the adaptive cerebellar controller infers a model from the error signal related to a sensorimotor input to produce effective corrective position and velocity terms. In this way, instead of propagating data from input to output as the forward architecture does, the recurrent architecture also propagates data from later processing stages to earlier ones. In the feedforward cerebellar control loop, the adaptive cerebellar module is embedded in the forward control loop and delivers add-on corrective torque values to compensate deviations in the base dynamics of the robotic arm model. The idealized correspondence with anatomical parts and processing functions is also indicated. (Bottom) Weight evolution in the cerebellar model manipulating different payloads operating with multiple plasticity mechanisms. Simulations were performed using plasticity at PF-PC, MF-DCN, and PC-DCN synapses and a custom-configured IO-DCN connection for manipulating 2 kg external payloads during 500 trials. The initial cerebellar system gain was properly set to operate with no payload. Evolution of the average error (MAE, black curve on the left) of the three robot joints during the learning process for 2 kg payload. The red curves on the left indicate the evolution of synaptic weight at the different synapses. Note that weights change rapidly at the beginning but then the cerebellar system works almost in open loop and no remarkable corrective action are applied by the cerebellar adapting system. PC and DCN neuron activity during a single trial show oscillations dictating the precise timing of force delivery to the joints in different trials. (Modified from Luque et al., 2011a, 2014).

When manipulating a tool, the cerebellar network acquires a dynamic and kinematic model of the tool. In this way, the manipulated tool becomes *de facto* as an extension of the arm allowing to perform accurate movements of the arm-object system as a whole. This unique capability is to a large extent based on the cerebellum sensory-motor integration properties. In order to establish a functional link between specific properties of neurons, network organization, plasticity rules and behavior, the cerebellar model needs to be integrated with a body (a simulated or real robotic sensory-motor system). Sensory signals need to be translated into biologically plausible codes to be delivered to the cerebellar network, and also cerebellar outputs need to be translated into representations suitable to be transferred to actuators (Luque et al., 2012). The experimental set-up is defined so as to monitor how accurately the system performs pre-defined movements when manipulating objects that significantly affect the arm/object kinematics and dynamics (Figure 7).

At this level, the cerebellar network is assumed to integrate sensory-motor signals by delivering corrective terms during movement execution (here a top-down approach is applied). In the framework of a biologically relevant task such as accurate object manipulation, different issues need to be addressed and defined by adopting specific working hypothesis and simplifications. For example: (i) PCs and DCN can be arranged in microcomplexes dealing with different degrees of freedom; (ii) error-related signal coming from the IO are delivered to PCs and drive learning at pf-PC synapses; (iii) neurons and connection can be simplified still maintaining the fundamental cerebellar network structure and functionality. There are different modeling approaches that have been simulated and tested (Luque et al., 2011a,b):

(1) Integrating the cerebellum in a feed-forward scheme delivering corrective terms to the spinal cord. In this case the cerebellum receives sensory inputs and produces motor corrective terms (the cerebellum implements an “inverse model”). Thus in this case the input and output representation spaces are different and the sensori-motor transformation needs to be performed also in the cerebellar network.
(2) Integrating the cerebellum in a feed-back (recurrent) scheme delivering corrective terms to the cerebellar cortex. In this case the cerebellum receives sensory-motor inputs and produces sensory corrective terms (the cerebellum implements a “forward model”; Kawato et al., 1988; Miyamoto et al., 1988; Gomi and Kawato, 1993; Yamazaki et al., 2015; Hausknecht et al., 2016).

Eventually, closed-loop robotic simulations allow to investigate the original issue of how the cerebellar microcircuit controls behavior in a novel manner. Here neurons and SNN are running in the robot. The challenge is clearly now to substitute the current simplified models of neurons and microcircuits with more realistic ones, so that from their activity during a specific behavioral task, the scientists should be able to infer the underlying coding strategies at the microscopic level.

## Current Perspectives for Realistic Cerebellar Modeling

On one hand, realistic cerebellar modeling is now advanced enough to generate predictions that may guide the subsequent search for critical physiological phenomena amongst the many that could be otherwise investigated. On the other hand, several new challenges await to be faced in terms of model construction and validation in order to explore physiological phenomena that have emerged from experiments. Realistic modeling is therefore becoming more and more an interactive tool for cerebellar research.

### Predictions of Realistic Cerebellar Modeling and their Experimental Testing

Cerebellar modeling is providing new opportunities for predicting biological phenomena that can be subsequently searched for experimentally. This procedure is relevant for several reasons. First, as discussed above, the computational models implicitly generate hypotheses providing the way for their subsequent validation or rejection. Secondly, the computational models can help focusing researcher’s interest toward specific questions. There are several examples that apply to different levels of cerebellar physiology.

In 2001, an advanced GrC model, based on the ionic conductance complement of the same neuron, predicted that a slow K current was needed to explain certain aspects of GrC firing and intrinsic GrC theta-band resonance. This current has been then looked for experimentally and its subsequent identification allowed to successfully complete the model and explain bursting and resonance in mechanistic terms (D’Angelo et al., 2001). In 2006, a mossy fiber-granule cell neurotransmission model, based on specific quantal release and receptor properties (Nieus et al., 2006), predicted that plasticity of intrinsic excitability could control rate coding while plasticity of release probability could control spike timing, as indeed verified experimentally. In 2007, a Golgi cell model actually predicted that Golgi cells were resonant in the theta-band a property that was then demonstrated experimentally (Solinas et al., 2007a,b). In 2007, a PC model predicted the coding properties of PCs in relation to LTD (Steuber et al., 2007). In 2009–2010 two models of the Golgi cell network predicted the impact of gap-junctions in regulating local GrC discharge and Golgi cell synchronization (Dugué et al., 2009; Vervaeke et al., 2010). In 2013, a theoretical article predicted that bidirectional plasticity had to exist at the mossy fiber—Golgi cell synapse (Garrido et al., 2013). This plasticity has subsequently been demonstrated (Locatelli et al., 2015). In 2014, a model including both excitatory and inhibitory neurotransmission predicted that phasic inhibitory mechanisms can dynamically regulate output spike patterns, as well as calcium influx and NMDA currents, at the mossy fiber-granule cell relay of cerebellum (Nieus et al., 2014). Again this prediction was accurately matched by the experiments. In 2015, a computational model predicted that the number of GrC dendrites that maximizes information transfer is actually coincident with that measured anatomically (Billings et al., 2014).

Yet other predictions are awaiting for experimental verification. In 2014, a closed-loop simulation predicted that cerebellar learning would accelerate toward biological levels if a form of mid-term plasticity would exist between the IO and DCN neurons (Luque et al., 2014). In 2016, another work predicted that STDP has the intrinsic capacity of binding learning to temporal network dynamics (Luque et al., 2016). Finally, very recently a mechanism of STDP learning involving the inhibitory interneuron network has been proposed (Garrido et al., 2016), that could be applicable to the GCL and explain how learning takes place in this cerebellar subnetwork. Thus, a new perspective for the near future is to extend the feed-back between computational models and experiments generating *de facto* a new powerful tool for cerebellar network investigation.

### New Challenges for Cerebellar Physiology and their Realistic Modeling

Amongst the new challenges that may benefit from enhanced and extended realistic models of the cerebellum, some have been highlighted in the present review and are summarized here.

There is a wealth of molecular and cellular phenomena, whose biological significance has been inferred experimentally, that could be incorporated into a realistic cerebellar model in order to investigate their implications for function. These include: the role of specific ionic channel properties in regulating neuronal excitation (amongst known examples see Jaeger et al., 1997; Bower and Beeman, 1998; Kubota and Bower, 2001; Ovsepian et al., 2013); the role of synaptic receptor properties in neuronal excitation and plasticity, like the voltage-dependence of NMDA receptor subtypes (Schwartz et al., 2012); the role of diffusible messengers like nitric oxide in coordinating long-term synaptic plasticity (Garthwaite, 2016); the role of intracellular biochemical cascades in the induction and expression of long-term synaptic plasticity (Tsukada et al., 1995; Schweighofer and Ferriol, 2000; Billings et al., 2014).

There are several properties of local microcircuits that are being discovered and that could be further understood by realistic cerebellar modeling. We have already mentioned the critical issue on how the cerebellum processes incoming information involving numerous molecular and cellular mechanisms that are only partially known. An issue that needs to be revisited, as it appears critical to understand the whole cerebellar functioning, is how the PC are activated by GrC through their aa (Gundappa-Sulur et al., 1999; Huang et al., 2006). Moreover, recent discoveries have opened new issues: ephaptic synapses have recently been revealed between basket cells (BCs) and PCs (Blot and Barbour, 2014), the connectivity of MLI involves complex spatial rules (Bower, 2010; Rieubland et al., 2014), the inhibitory network in the cerebellar granular layer involves gap junctions and reciprocal inhibitory synapses (Dugué et al., 2009; Szoboszlay et al., 2016; van Welie et al., 2016), the inferior olivary neurons are connected through gap junctions (Rothman et al., 2009; Rancz and Häusser, 2010; Lefler et al., 2014).

There are aspects of intracerebellar organization and connectivity that remain to be incorporated into large-scale realistic models, including the granular layer-molecular layer projections (Valera et al., 2016), the PC-DCN convergence (Person and Raman, 2012b), the DCN-granular layer projections (Houck and Person, 2015), the PC-DCN-IO loops (Libster and Yarom, 2013). Beyond this, these are needed for guided cerebellar model simplification and incorporation into large-scale networks running into robotic controllers and simulated environments (Garrido et al., 2013; Casellato et al., 2015; Yamazaki et al., 2015).

On the pathophysiological side (Chen et al., 2010; Libster et al., 2010; Ovsepian et al., 2013; Kros et al., 2015), there is a wealth of hypothesis that have or would benefit of realistic modeling. Ataxia has long been attributed to cerebellar dysfunction. Recently, several ionic channel and neuronal alterations have been linked to ataxia (Libster et al., 2010) and to the disruption of dynamics in the olivo-cerebellar circuit (Chen et al., 2010). There are specific properties of the cerebellar output that are critical for controlling extracerebellar networks and their pathological states, like in cebro-cortical spike-and-wave discharge (e.g., see Ovsepian et al., 2013; Kros et al., 2015). This kind of observations may provide critical test-benches for realistic model validation and prediction.

Finally, in perspective, the connectivity of the cerebellar network in long-range loops appears to be critical to understand microcircuit functions. Following the fundamental recognition of its involvement in sensory-motor coordination and learning, the cerebellum is now also believed to take part in the processing of cognition and emotion (Schmahmann, 2004) by exploiting the connectivity of the cerebellar modules with specific brain structures through different cerebro-cerebellar loops. It has been proposed that a similar circuit structure in all cerebellar areas may carry out various operations using a common computational scheme (D’Angelo and Casali, 2013). Since there is an intimate interplay between timing and learning at the cellular level that is reminiscent of the “timing and learning machine” capabilities long attributed to the cerebellum, it is conceivable that realistic models developed for sensori-motor control might also apply to cognitive-emotional control once integrated into the appropriate loops.

## A Manifesto for Collaborative Cerebellar Modeling

This review has summarized some relevant aspects characterizing the cerebellar circuit showing how these have been conceptualized and modeled. Still, there are several issues that deserve attention, ranging from molecular to neuronal, microcircuit, macrocircuit and integrative aspects, and even more it is clear that all these aspects are tightly bound. There is no solution through a single experiment or model, so that understanding the *structure-function-dynamics* relationship of the cerebellum requires a continuous bottom-up top-down dialog (Akemann et al., 2009).

Realistic modeling is now opening new perspectives. The main challenge is to join precise network wiring with accurate representations of neuronal and synaptic properties in order to be able to simulate local network dynamics. The introduction of synaptic and non-synaptic plasticity in its various forms and locations could then allow to understand how input patterns can reconfigure the network during ontogenetic development and in the mature state. Finally, full exploitation of cerebellar network capabilities would require simulations operated in closed-loop in robotic systems. It is envisaged that such systems will be able in the future to emulate physiological and pathological states, providing the basis for protocols of network-guided robotic neurorehabilitation.

Large-scale simulations running efficiently on supercomputers are now possible, and software development systems have been designed and tested (Bhalla et al., 1992; Hines and Carnevale, 1997; Bower and Beeman, 2007; Gleeson et al., 2007, 2010; Davison et al., 2009; Hines et al., 2009; Cornelis et al., 2012a). While this may be sufficient for elaborating complex codes in an iterative reconstruction/validation process, simulating network adaptation during learning would require several repetitions over prolonged time periods. In this scenario, a large-scale cerebellar network embedding synaptic learning rules should be running inside a whole sensory-motor control system generating a massive computational load and leading to unaffordable simulation times. To this aim, efficient codes have been developed (Eppler et al., 2008; Bednar, 2009; Zaytsev and Morrison, 2014). The problem that remains will be that of providing efficient model simplifications still maintaining the salient computational properties of the network (e.g., see the chapter above Casellato et al., 2012, 2014, 2015; Garrido et al., 2013; Luque et al., 2014). Eventually, neuromorphic hardware platforms will have to be considered for the cerebellum as well as for the cerebral cortex (Pfeil et al., 2013; Galluppi et al., 2015; Lagorce et al., 2015). It can be envisaged that realistic modeling of the cerebellum, with the reconstruction of neurons and large-scale networks based on extended data-sets and running on supercomputing infrastructures, will require a world-wide collaborative effort as it has been proposed for other brain structures like the neocortex and hippocampus (Markram, 2006; Cornelis et al., 2012a; Crook et al., 2012; Kandel et al., 2013; Bower, 2015; Ramaswamy et al., 2015).

## Author Contributions

ED’A coordinated and wrote the article helped by all the other authors.

## Conflict of Interest Statement

The authors declare that the research was conducted in the absence of any commercial or financial relationships that could be construed as a potential conflict of interest.

## Abbreviations

aa: ascending axon
APN: anterior pontine nucleus
ATN: anterior thalamic nuclei
BC: basket cell
BG: basal ganglia
cf: climbing fiber
Ca^2+^: calcium ions
cGMP: cyclic GMP
DCN: deep cerebellar nuclei
DAG: diacyl-glycerol
GoC: Golgi cell
glu: glutamate
GC: guanyl cyclase
GCL: granular cell layer
GrC: granule cell
IO: inferior olive
IP3: inositol-triphosphate
LC: Lugaro cell
ML: molecular layer
MLI: molecular layer interneuron
mf: mossy fiber
MC: motor cortex
NO: nitric oxide
NOS: nitric oxide synthase
PKC: protein kinase C
pf: parallel fiber
PC: Purkinje cell
PC: parietal cortex
PIP: phosphatidyl-inositol-phosphate
PFC: prefrontal cortex
PCL: Purkinje cell layer
RN: reticular nucleus
SC: stellate cell
TC: temporal cortex
STN: subthalamic nucleus
UBC: unipolar brush cell.
